# The Effects of a Psychomotor Training Program on Physical Coordination in Children with Development Delay

**Published:** 2017-06

**Authors:** Do-Jin KIM, Jong-Hyuck KIM, Wi-Young SO, Eun-Ju CHOI

**Affiliations:** 1. Dept. of Rehabilitation Sports, Bucheon University, Bucheon-Si, Korea; 2. Dept. of Beauty & Health, Jungwon University, Goesan-Gun, Korea; 3. Sports and Health Care Major, Korea National University of Transportation, Chungju-Si, Korea; 4. Division of Sport Sciences, Konkuk University, Chungju-Si, Korea

## Dear Editor-in-Chief

Social interaction plays an important role in forming independent social skills and early social relationship. It also affects other developmental fields such as language and communication (1, 2). Studies on psychomotor training for social skills or social interaction are ongoing (3, 4).

Psychomotor training enables children to change play through communication and interaction with other children, develop roles and rules for the self, develop an understanding of self and others, improve their communication ability and socialization skills through the process of finding pleasure, and develop self-control. It also helps to form a healthy personality through personal character-oriented behavior experience by breaking away from a merit system that operates on fault orientation and focuses on the weakness, abnormal behavior, and shortcomings of children (3, 4). Children are usually active and expressive when they are free without being forced to play (5). The training allows the individual interests of children to develop, induces curiosity, supports the desire to obtain new experiences, and supports variations in children’s behavior. This training consists of experience-based behavior tasks that enable children to realize that they are important members’ in-group activities and helps them to form positive self-concepts and experience that they can exert their influence (3–5).

This study aimed to provide basic data regarding various psychomotor physical activity programs for children with developmental delay in the adapted physical education field by applying for a 12-wk psychomotor physical activity program in children with developmental delay in 2016.

The subjects were 15 boys with developmental delay who were 5–7 yr old, lived in Seoul, visited M Children Development center, and were diagnosed with developmental delay through medical tests. The subjects were classified into the experimental group (n=8; age, 5.75±0.70 yr; height, 115.30±2.55 cm; weight, 22.36±2.13 kg) and control group (n=7; age, 5.86±0.90 yr; height, 116.25±3.18 cm; weight, 22.91±2.54 kg) boys to compare the effects of the intervention program. The psychomotor training program was conducted twice a week for 60 min over 12 wk. This program aims to inculcate confidence in children by making them participate voluntarily in various physical, material, and social experiences. This study applied basal fitness, obstacle running, and prop play. All participants and their parents submitted written consent forms. The details of the participants are shown in Table 1.

**Table 1:** Psychomotor physical activity program

**Weeks**	**Experimental process**	**Subject**	**Program**
1–4 wk	Trial and search	Basal fitness	Gross-motor, fine motor stretching, jumping with music, two legs/one leg jumping, hoop pass, balancing gym ball
5–8 wk	Experimental stage	Obstacle running	Parachute running, balance beam, steps, trampoline, ladder, mat running
9–12 wk	Autonomous stage	Props play	Mini basketball, ball-pool play, mini billiard, mini fishing

The KTK (Körperkoordinationstest für Kinder) is one such test that was invented and standardized by German scholars Kiphard & Schilling (6); it is intended to test motor function of children from 5–14 yr old. The objects in the test were backward balance (motor quotient 1; MQ1), which consists of nine turns stepping forward and backward by balancing on a 6 cm wide balance beam. One leg jump (motor quotient 2; MQ2) consists of jumping over a sponge 1.5 m high and wide; the subject has to jump twice with the same leg and 3 turns are allowed. Left and right jump (motor quotient 3; MQ3) consists of jumping to the left and right across a stick; 2 turns are permitted. Moving on one side (motor quotient 4; MQ4) consists of moving 3–4 m and maintaining a foothold for 20 sec; 2 trials with 10 sec rest are allowed.

All results are presented as mean±standard deviation. The KTK physical cooperation test was conducted before and after the psychomotor program and repeated measure, analysis of variance was conducted. Statistical significance was set at *P*<0.05 and all analyses were performed by using SPSS ver. 18.0 (SPSS, Chicago, IL, USA). The changes in motor function after 12 wk of psychomotor physical activity program are shown in Table 2. Statistically, significant differences were seen in all variables: backward balancing (MQ1), one leg jump (MQ2), left and right jump (MQ3), and moving on one side (MQ4) (*P*≤0.006). The psychomotor training program brought about a significant change in the children with developmental delay in the experimental group.

**Table 2:** Changes in motor function after 12 wk of the psychomotor training program

**Categories**	**Exercise**	**Pre-test**	**Post-test**	**Interaction (Group×Time)**	
				***F***	***P***
Backward step balancing	EXG	72.37±17.54	87.50±15.56	20.664	0.001^**^
(MQ1; score)	COG	76.14±13.64	77.57±11.14		
One leg jump	EXG	80.00±23.43	94.37±18.77	19.055	0.001^**^
(MQ2; score)	COG	78.00±15.56	78.71±12.56		
Left and right jump	EXG	75.62±27.50	92.37±24.77	10.985	0.006^**^
(MQ3; score)	COG	79.14±21.60	82.42±18.10		
Moving on one side	EXG	64.00±13.96	83.87±13.24	61.133	<0.001^***^
(MQ4; score)	COG	70.28±16.37	72.14±14.20		

MQ; Motor quotient, EXG; experimental group, COG; control group

** *P*<0.01,

*** *P*<0.001; tested by repeated measure analysis of variance
